# Twenty-Five Years of Firearm Homicides and Suicides Among US Children and Young Adults, 1999 to 2024: Intersectional Surveillance Analysis

**DOI:** 10.2196/96931

**Published:** 2026-07-31

**Authors:** Chuka Emezue, Tipparat Udmuangpia

**Affiliations:** 1 Women, Children, and Family Nursing, College of Nursing, Rush University, 600 S Paulina St, Chicago, IL, 60612, United States, 1 3129416151; 2 Boromarajonani College of Nursing, Khon Kaen, Faculty of Nursing, Praboromarajchanok Institute, Nonthaburi, Thailand

**Keywords:** firearm mortality, youth, racial disparities, health equity, intersectionality, homicide, suicide, surveillance, epidemiology, CDC WONDER, Centers for Disease Control and Prevention

## Abstract

**Background:**

Firearm mortality surveillance relies on aggregate demographic categories or low-dimensional stratifications, obscuring how race, ethnicity, sex, age, and intent intersect to shape risk among youth and young adults. The Centers for Disease Control and Prevention (CDC)’s transition to single-race population estimates created a structural break between historical and contemporary data series. Consequently, the 25-year trajectory of intersectional firearm mortality across changing federal classification frameworks remains uncharacterized.

**Objective:**

We aimed to characterize 25-year national trajectories in firearm homicide and suicide across intersecting domains of race or ethnicity, sex, age, and intent among US youth and young adults (aged 0‐24 y), identifying specific sociodemographic groups experiencing the highest and most rapidly changing burdens.

**Methods:**

This study used the CDC WONDER Underlying Cause of Death database (25-year window from 1999‐2024). To address the 2018 transition, a 2-period design was deployed: period 1 (1999 to 2020) used bridged 4-category race data, and period 2 (2018 to 2024) used 6-category single-race data. Crude death rates and disparity ratios (referenced to non-Hispanic White youth) were calculated using the Byar approximation. Average annual percentage changes (AAPCs) were modeled via log-linear regression. Analyses incorporated finalized 2024 CDC WONDER data in January 2026.

**Results:**

Among 200,704 firearm deaths among youth aged 0‐24 years from 1999 to 2024, overall mortality declined through 2013, reversed in 2014, and rose 28.9% from 2019 to 2020. In 2024, the overall rate declined 12% from 2023 but remained above the 2019 prepandemic baseline. During 2018‐2024, Black non-Hispanic male youth experienced the highest pooled firearm homicide rate (44.03 per 100,000; 95% CI 43.46‐44.61), 23.8 times the rate among White non-Hispanic males. The annual rates within this cohort increased from 33.28 in 2018 to 53.75 in 2021 before declining to 37.06 in 2024 (AAPC, +2.4%; *P*=.56), while the Black-to-White disparity ratio widened from 19.3 to 24.4. Black non-Hispanic female youth had the highest female firearm homicide rate (6.03 per 100,000), exceeding male rates in 7 of 12 other demographic groups. American Indian or Alaska Native non-Hispanic male youth experienced the highest pooled firearm suicide rate (11.74 per 100,000). Firearm suicide increased significantly among Black non-Hispanic male youth (AAPC, +9.2%; 95% CI +3.2% to +15.4%; *P*=.01), with a substantial but nonsignificant upward trend among Black non-Hispanic female youth (AAPC, +11.7%; *P*=.08), while White non-Hispanic male rates remained stable (AAPC, −0.1%; *P*=.91). Consequently, the Black-to-White non-Hispanic male suicide rate ratio shifted from 0.67 in 2018 to 1.07 in 2024, marking the first time Black youth rates surpassed White youth rates in modern public health surveillance history.

**Conclusions:**

Firearm homicide and suicide among US youth exhibit distinct demographic concentration patterns that are systematically obscured by aggregated public health surveillance. Disaggregated surveillance remains imperative to design targeted prevention strategies.

## Introduction

Firearm injury has emerged as a leading cause of death among children, adolescents, and young adults aged 1 to 24 years in the United States [1], surpassing motor vehicle crashes, cancer, and all other causes of mortality in recent years [2]. Within this composite burden, firearm homicides and suicides function as 2 epidemiologically distinct phenomena [1]. While both public health crises share upstream structural and social determinants, they diverge in their sociodemographic concentrations, proximal mechanisms, case-fatality rates, geographic distribution, and intervention entry points [3-6]. Two decades of national surveillance have structured prevention science and federal investment around a seemingly stable race-by-intent dichotomy. This paradigm is characterized by disproportionately high firearm homicide rates documented among non-Hispanic Black youth and elevated firearm suicide mortality among non-Hispanic White and American Indian or Alaska Native (AI/AN) youth [1,7-12]. While this framing has clarified the population-level magnitude of firearm injury among these groups, it has also concealed the intersectional gradients within and across other aggregated populations.

National surveillance reports rarely stratify age, sex, race or ethnicity, and injury intent jointly. Where such multiway stratifications are produced in subnational data, strict federal data suppression policies systematically suppress cells containing fewer than 10 deaths to protect privacy [13]. At the national level, the fundamental constraint is not the technical capacity of the underlying surveillance infrastructure, which permits 4-way disaggregation via the Centers for Disease Control and Prevention (CDC) WONDER database, but the reporting conventions that govern what enters published epidemiological records. Consequently, contemporary shifts in firearm violence and injury risk levels among AI/AN, Native Hawaiian or Other Pacific Islander (NH/OPI), multiracial, and non-Hispanic Black adolescent female populations remain underrepresented in standard surveillance literature.

Recent intersectional analyses have started to address these gaps. Mariño-Ramírez et al [9] stratified pediatric firearm mortality by race and ethnicity across the years 1999‐2020 and called for more granular reporting. Likewise, Lee et al [10] simultaneously stratified by age, sex, race, and ethnicity through 2023 and documented the intersections of firearm and motor vehicle mortality; while Klein-Cloud et al [13] examined racial and ethnic disparities through 2023, raising the data suppression and classification concerns examined further in the present analysis.

However, 3 critical limitations of the current literature remain. First, the 2018 CDC transition from a bridged 4-category race classification scheme to a 6-category single-race scheme has effectively bifurcated the literature into isolated pre-2020 and post-2018 analyses, foreclosing an examination of the longitudinal arc of youth firearm mortality across the classification transition. Additionally, emerging intersectional signals remain underexamined relative to their epidemiological significance; notably, the recent convergence of firearm suicide mortality between non-Hispanic Black and White male youth, and the elevated firearm homicide burden among non-Hispanic Black adolescent and young adult female youth. Finally, this analysis incorporates finalized 2024 mortality data, released by the National Center for Health Statistics (NCHS) in January 2026. This additional year allows examination of whether pandemic-era increases in youth firearm mortality continued, stabilized, or declined.

Intersectionality frameworks, as articulated by Crenshaw [14] and adapted for population health by Bowleg [15] and Bauer [16], emphasize that social positions related to race, gender, class, and other axes are occupied simultaneously rather than additively, producing patterns of risk that are not visible when each axis is examined independently or only through two-dimensional comparisons. Applied to injury epidemiology, an intersectional surveillance lens directs analytic attention to specific demographic groups whose risks may be obscured when surveillance data are stratified by only 1 or 2 characteristics at a time. For example, the firearm mortality burden among Black non-Hispanic adolescent girls or AI/AN non-Hispanic young adult males may be less visible in race-only or sex-only analyses. Although vital statistics cannot directly measure the structural processes that produce these inequities, intersectionally disaggregated surveillance can identify where mortality is concentrated and help guide subsequent etiologic, policy, and prevention efforts [16,17].

This study examines national youth firearm mortality in the United States from 1999 through 2024 using the CDC WONDER Underlying Cause of Death database. To accommodate the federal classification transition, we adopt a 2-period analytic design. Period 1 (1999 to 2020) draws on the bridged-race data to characterize the 25-year temporal arc, while period 2 (2018 to 2024) leverages the single-race dataset to estimate contemporary intersectional patterns at a higher demographic resolution. The overlapping years (2018 to 2020) function as an internal consistency check to verify rate stability across both data structures rather than as an analytic break point. This design contributes to the current literature as it situates youth firearm mortality within the first complete year of postpandemic vital statistics; it treats the bridged-to-single-race transition as an analytic feature rather than truncating estimation at a single classification era, and it isolates several critical, underexamined intersectional signals across race or ethnicity, sex, age, and injury intent.

## Methods

### Study Design and Data Source

We conducted a serial cross-sectional, population-based descriptive analysis of firearm deaths among US children, adolescents, and young adults aged 0 to 24 years between 1999 and 2024, using the CDC WONDER Underlying Cause of Death database. WONDER compiles death certificate records through the National Vital Statistics System and provides annual counts, population denominators, and crude rates by demographic and cause-of-death categories [18]. Population denominators were drawn from CDC WONDER bridged-race population estimates for period 1 (1999 to 2020) and single-race population estimates for period 2 (2018 to 2024). The analytic population was restricted to US residents of the 50 states and the District of Columbia.

The current study population was defined as individuals aged 0 to 24 years, comprising early childhood (0 to 12 y), adolescence (13 to 17 y), and young adulthood (18 to 24 y), consistent with established developmental and public health frameworks [19,20]. This upper boundary extends beyond the 0-to-17-year range commonly used in pediatric firearm surveillance reports [2,7,21], which exclude young adults aged 18 to 24 years, a developmental demographic that bears a disproportionate share of the national firearm homicide burden, particularly among Black and Hispanic male youth [3,21].

The 25-year analytic window was bounded by historical and administrative considerations of data continuity. The lower bound of 1999 represents the first full calendar year in which the United States implemented the *ICD-10* (*International Statistical Classification of Diseases, Tenth Revision*) for underlying cause-of-death coding, and the first year for which CDC WONDER bridged-race population denominators are available [18,22]. The upper bound of 2024 incorporates the most recently finalized national mortality file released by the NCHS in January 2026 [18]. The window spans 25 years, from 1999 through 2024, and comprises 26 annual observations. This window enables the contextualization of contemporary, postpandemic shifts within a quarter-century historical trajectory.

### Ethical Considerations

This analysis used deidentified, publicly available, aggregated vital statistics data and was therefore determined not to constitute human participants research as defined under 45 CFR (Code of Federal Regulations) §46.102. Institutional review board review was not required under institutional policy governing the secondary analysis of public-use, fully anonymized federal data, and no application was submitted. The CDC WONDER public-use data files contain no direct or indirect personal identifiers. NCHS suppresses cells with fewer than 10 deaths, including finely stratified national cells, to protect confidentiality. The present analysis used visible cells and explicitly identified suppressed and statistically unreliable estimates that satisfied federal analytic reliability thresholds, with unstable rates explicitly flagged. As the underlying records are fully anonymous and collected via routine statutory vital statistics registration, informed consent was not applicable.

### Firearm Fatality Classification

Firearm fatalities were identified using *ICD-10* underlying cause-of-death codes. In this analysis, the term “intent” refers to the manner of death recorded on the death certificate, representing the standard NCHS classification of the circumstances surrounding the fatal injury. Five mutually exclusive intent categories were retained: firearm homicide (X93-X95); firearm suicide (X72-X74); unintentional firearm injury, defined as a fatal accidental discharge of a firearm rather than a category of homicide or suicide (W32-W34); firearm injury of undetermined intent (Y22-Y24); and legal intervention involving a firearm (Y35.0).

Firearm homicide and suicide served as the primary intent categories for all intersectional analyses. Unintentional, undetermined, and legal intervention deaths were retained in cumulative *all-cause* totals but were excluded from intent-specific trend estimation, given high suppression rates across multiway demographic and intersectional strata. Cumulative 25-year death totals were calculated by aggregating all period 1 deaths (1999‐2020) and adding unique deaths from the nonoverlapping years of period 2 (2021‐2024) to avoid duplication. All 2024 data reflect finalized counts released by CDC WONDER in January 2026 [18].

### Two-Period Analytic Design

In data year 2021, the CDC fully transitioned its multiple-cause-of-death files from a bridged 4-category race classification scheme (White, Black, AI/AN, and Asian or Pacific Islander, with multiple-race decedents algorithmically assigned to a single category) to a 6-category single-race scheme that separately identifies Asian, NH/OPI, and multi-racial decedents [22]. The legacy bridged-race data preserves long-term trend comparability back to 1999 but obscures contemporary diversity, whereas the single-race file allowed for greater contemporary sociodemographic comparability (back to 2018) but cannot be extended to the prebridging era. As these 2 population denominator systems introduce a structural trend break, a single continuous analytic framework spanning 1999 to 2024 was computationally untenable.

Accordingly, race and ethnicity were combined into a single mutually exclusive variable for all analyses (eg, non-Hispanic Black and Hispanic). The overlapping years (2018 to 2020) were used as an internal validation window to verify rate consistency and mathematical stability across both classification methods. The descriptive simultaneous stratification by race or ethnicity, sex, age, and injury intent used in this study constitutes a surveillance application of intersectional theory to isolate overlooked risk concentrations, rather than a formal quantitative intersectional decomposition [16].

### Statistical Analysis

Crude firearm death rates per 100,000 population were calculated for each demographic subgroup. Crude rather than age-adjusted rates were prioritized because simultaneous multiway stratification renders within-stratum age adjustment statistically volatile and analytically untenable for low-denominator populations, such as AI/AN, NH/OPI, and multiracial youth [9,21,23]. Disparity ratios were computed using non-Hispanic White males as the reference group for male comparisons and non-Hispanic White females for female comparisons; absolute crude rates were also compared directly across sex lines where female burdens eclipsed male baselines. Further, 95% CIs for rates and ratios were derived using the Byar approximation, which provides robust coverage for the small expected cell counts characteristic of fine-grained intersectional strata [24].

Cells based on fewer than 20 deaths were flagged as analytically unreliable per NCHS standards. Within the primary pooled-age contemporary intersectional matrix for period 2 (6 racial groups x 2 Hispanic-origin categories x 2 sexes x 3 primary intent categories [homicide, suicide, and unintentional injury]), there were 72 distinct cells. Of these, 19 of 72 (26.4%) cells had counts fewer than 10 and were omitted from detailed stratification, and 11 of 72 (15.3%) cells were flagged as unreliable, yielding fully stable estimates for 42 of 72 (58.3%) baseline cells. Rate instability was heavily concentrated among NH/OPI youth (10/12, 83.3% cells) and within specific unintentional injury strata.

To characterize directional trends within period 2 (2018-2024), average annual percentage changes (AAPCs) were estimated by log-linear regression of the natural logarithm of the annual crude rate on calendar year. These were calculated separately for firearm homicide and suicide across non-Hispanic race-sex strata that demonstrated 3 or more reliable annual observations. The AAPC was calculated as (exp[*β*] − 1)×100, where *β* is the estimated slope parameter. Further, 95% CIs were derived from the t-distribution of *β* with n − 2 degrees of freedom. Statistical significance was assessed using 2-sided *t* tests with an a priori alpha =.05. As this study’s period encompasses a nonlinear post-2020 mortality surge and subsequent decline, the log-linear AAPC is explicitly interpreted as the net annualized trend velocity across the interval rather than a monotonic trajectory. All analyses were executed in Python (version 3.11; Python Software Foundation) using the Pandas, NumPy, SciPy, Matplotlib, and Seaborn libraries. This study adhered strictly to the STROBE (Strengthening the Reporting of Observational Studies in Epidemiology) reporting guideline; see Checklist 1.

## Results

### Cumulative Burden and Temporal Trends, 1999-2024

**Figure 1.**
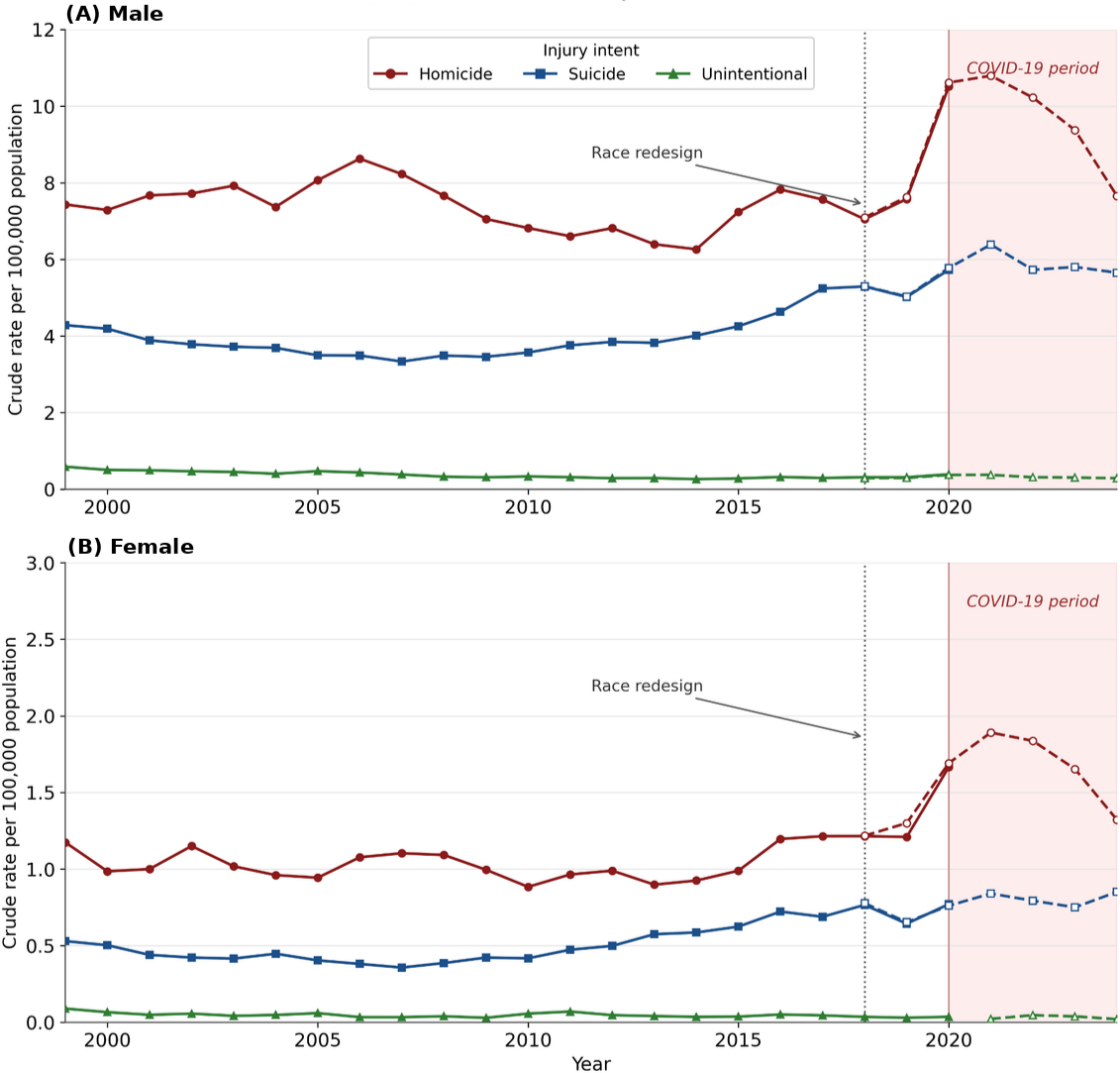
Crude firearm mortality rates among US youth and young adults aged 0 to 24 years, by injury intent and sex, 1999‐2024. Homicide, suicide, and unintentional intent are shown as separate trend lines. The shaded region indicates the elevated mortality period of 2020‐2024. The vertical dashed line at 2018 marks the CDC transition from bridged to single-race classification; dotted lines across 2018‐2020 indicate overlapping years and do not represent a true mortality trend. Across the 2018 to 2020 overlap, the 2 periods nearly coincide, and any residual difference reflects the change in population denominators between the bridged-race and single-race files rather than a true change in mortality, so rates from the two periods are not directly compared. Source: CDC WONDER [25]. CDC: Centers for Disease Control and Prevention.

From 1999 through 2024, a total of 200,704 firearm deaths occurred among US youth and young adults aged 0 to 24 years (period 1: 161,313; period 2, nonoverlapping years 2021‐2024: 39,391). Firearm mortality remained concentrated among male youth throughout the 25-year period, though the female share of the burden rose modestly, from 11.4% (18,459/161,313) of deaths in period 1 to 13% (8543/65,472) in period 2, even as the sex-specific trajectories diverged by intent (Figure 1). Across the full 25-year window, overall crude firearm mortality declined steadily from 1999 through 2013, reversed direction in 2014, and accelerated sharply during the pandemic period, rising 28.9% between 2019 and 2020 (from 7.69 to 9.91 per 100,000). Rates remained elevated through 2022 before partially stabilizing in 2023 and declining in 2024 (Figure 1). The recent 2024 overall rate of 8.24 per 100,000 represented a 12% decrease from 2023, the largest single-year decline observed during this 25-year period, though overall youth firearm mortality remained above the 2019 prepandemic baseline.

When examined by intent, firearm homicide and firearm suicide followed divergent trajectories within this aggregate trend. Among Black non-Hispanic male youth, homicide rates rose from 33.44 per 100,000 in period 1 to 44.03 per 100,000 in period 2, and Black non-Hispanic female homicide rates nearly doubled from 3.46 to 6.03 per 100,000 (Tables 1 and 2). Firearm suicide rates among AI/AN non-Hispanic male youth rose from 8.47 to 11.74 per 100,000 across the 2 periods, and Black non-Hispanic male suicide rates rose from 3.51 to 6.33 per 100,000. The 2 periods nearly coincide across the 2018 to 2020 overlap in Figure 1. The small residual differences reflect changes in population denominators between the bridged-race and single-race files rather than true changes in mortality; therefore, rates were not directly compared across periods.

**Table 1.** Crude firearm mortality rates per 100,000 by race, Hispanic origin, sex, and injury intent, US youth ages 0‐24 years, and years 1999‐2020 (bridged-race classification). CIs were calculated using the Byar approximation.

Race/ethnicity group	Sex	Homicide rate (95% CI)	Suicide rate (95% CI)	Unintentional rate (95% CI)
Black or African American				
Non-Hispanic	Female	3.46 (3.37‐3.55)	0.36 (0.33‐0.39)	0.09 (0.07‐0.10)
Non-Hispanic	Male	33.44 (33.16‐33.72)	3.51 (3.42‐3.60)	0.73 (0.69‐0.77)
Hispanic	Female	0.70 (0.56‐0.86)	Unreliable^a^	Suppressed^b^
Hispanic	Male	4.49 (4.14‐4.85)	0.92 (0.76‐1.08)	Unreliable
White				
Non-Hispanic	Female	0.58 (0.56‐0.60)	0.67 (0.65‐0.69)	0.04 (0.04‐0.05)
Non-Hispanic	Male	1.59 (1.56‐1.62)	5.33 (5.28‐5.39)	0.35 (0.34‐0.37)
Hispanic	Female	0.95 (0.91‐0.99)	0.28 (0.26‐0.30)	0.03 (0.02‐0.03)
Hispanic	Male	8.21 (8.09‐8.33)	2.39 (2.33‐2.46)	0.25 (0.23‐0.27)
American Indian or Alaska Native				
Non-Hispanic	Female	1.28 (1.07‐1.49)	1.08 (0.89‐1.27)	Unreliable
Non-Hispanic	Male	6.19 (5.74‐6.65)	8.47 (7.93‐9.01)	0.87 (0.70‐1.04)
Hispanic	Female	Unreliableᵃ	Unreliable	Suppressed
Hispanic	Male	Suppressed	Suppressed	Suppressed
Asian or Pacific Islander				
Non-Hispanic	Female	0.39 (0.34‐0.44)	0.29 (0.25‐0.33)	Suppressed
Non-Hispanic	Male	2.03 (1.91‐2.14)	1.74 (1.63‐1.84)	0.09 (0.07‐0.12)
Hispanic	Female	Suppressed	Unreliable	Suppressed
Hispanic	Male	1.59 (1.24‐2.01)	0.94 (0.68‐1.27)	Suppressed

a Unreliable: fewer than 20 deaths; interpret with caution. Source: CDC (Centers for Disease Control and Prevention) WONDER [25].

b Suppressed: fewer than 10 deaths. Source: CDC (Centers for Disease Control and Prevention) WONDER [25].

**Table 2.** Crude firearm mortality rates per 100,000 population pooled 2018-2024, 6-category single-race classification per 1997 OMB^a^ standards. CIs were calculated using the Byar approximation.

Race/ethnicity group	Sex	Homicide rate (95% CI)	Suicide rate (95% CI)	Unintentional rate (95% CI)
Black or African American				
Non-Hispanic	Female	6.03 (5.82‐6.25)	0.87 (0.79‐0.95)	0.14 (0.10‐0.17)
Non-Hispanic	Male	44.03 (43.46‐44.61)	6.33 (6.11‐6.55)	0.86 (0.78‐0.94)
Hispanic	Female	1.03 (0.77‐1.35)	0.35 (0.21‐0.55)	Suppressed^b^
Hispanic	Male	6.71 (6.01‐7.40)	2.30 (1.89‐2.70)	0.21 (0.10‐0.37)
White				
Non-Hispanic	Female	0.65 (0.61‐0.69)	0.95 (0.90‐0.99)	0.04 (0.03‐0.05)
Non-Hispanic	Male	1.85 (1.79‐1.92)	7.01 (6.89‐7.13)	0.30 (0.27‐0.32)
Hispanic	Female	1.20 (1.13‐1.28)	0.50 (0.45‐0.55)	0.02 (0.01‐0.03)
Hispanic	Male	7.33 (7.14‐7.52)	3.70 (3.57‐3.84)	0.22 (0.19‐0.26)
American Indian or Alaska Native				
Non-Hispanic	Female	2.08 (1.59‐2.68)	1.77 (1.32‐2.33)	Suppressed
Non-Hispanic	Male	9.29 (8.20‐10.38)	11.74 (10.51‐12.97)	1.14 (0.79‐1.59)
Hispanic	Female	0.40 (0.21‐0.71)	0.40 (0.21‐0.71)	Suppressed
Hispanic	Male	1.93 (1.47‐2.49)	1.61 (1.19‐2.12)	Suppressed
Asian				
Non-Hispanic	Female	0.26 (0.19‐0.34)	0.43 (0.35‐0.53)	Suppressed
Non-Hispanic	Male	1.11 (0.97‐1.26)	2.41 (2.20‐2.63)	0.06 (0.03‐0.11)
Hispanic	Female	Suppressed	Suppressed	Suppressed
Hispanic	Male	1.63 (0.96‐2.57)	2.17 (1.39‐3.23)	Suppressed
Native Hawaiian or Other Pacific Islander				
Non-Hispanic	Female	Suppressed	1.35 (0.65‐2.48)	Suppressed
Non-Hispanic	Male	8.34 (6.44‐10.64)	5.13 (3.67‐6.99)	Suppressed
Hispanic	Female	Suppressed	Suppressed	Suppressed
Hispanic	Male	2.62 (1.25‐4.81)	3.14 (1.62‐5.48)	Suppressed
More than 1 race				
Non-Hispanic	Female	1.02 (0.86‐1.18)	0.50 (0.39‐0.63)	Suppressed
Non-Hispanic	Male	3.40 (3.12‐3.69)	3.28 (3.00‐3.57)	0.15 (0.09‐0.22)
Hispanic	Female	0.73 (0.48‐1.05)	0.49 (0.30‐0.77)	Suppressed
Hispanic	Male	3.22 (2.66‐3.77)	2.25 (1.81‐2.76)	Suppressed

a OMB: Office of Management and Budget.

b Suppressed: fewer than 10 deaths. Source: CDC (Centers for Disease Control and Prevention) WONDER [25].

### Firearm Homicide Rates by Race or Ethnicity and Sex, 2018-2024

#### Male Homicides

**Figure 2.**
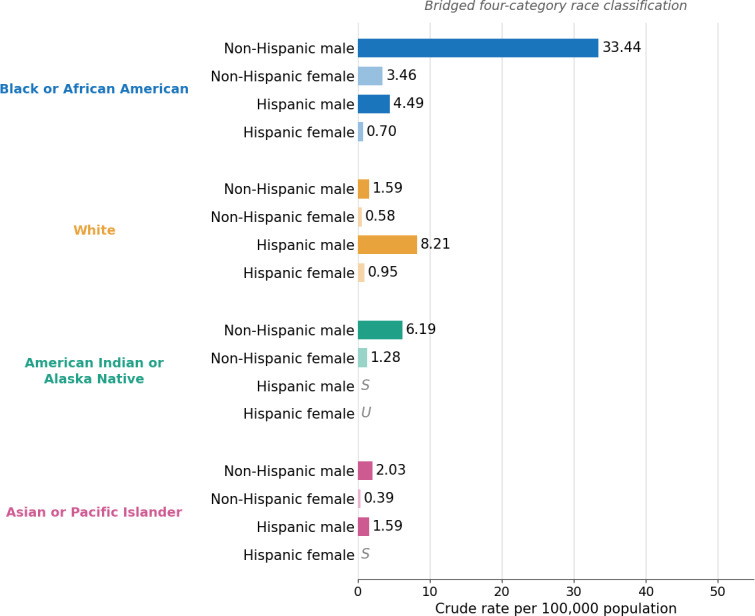
Crude firearm homicide rates among US youth and young adults aged 0 to 24 years, by race, Hispanic ethnicity, and sex, using the bridged 4-category race classification for 1999 to 2020. S indicates suppressed data because the cell contained fewer than 10 deaths; U indicates unreliable data because the estimate was based on fewer than 20 deaths. Source: CDC WONDER [25]. CDEC: Centers for Disease Control and Prevention.

**Figure 3.**
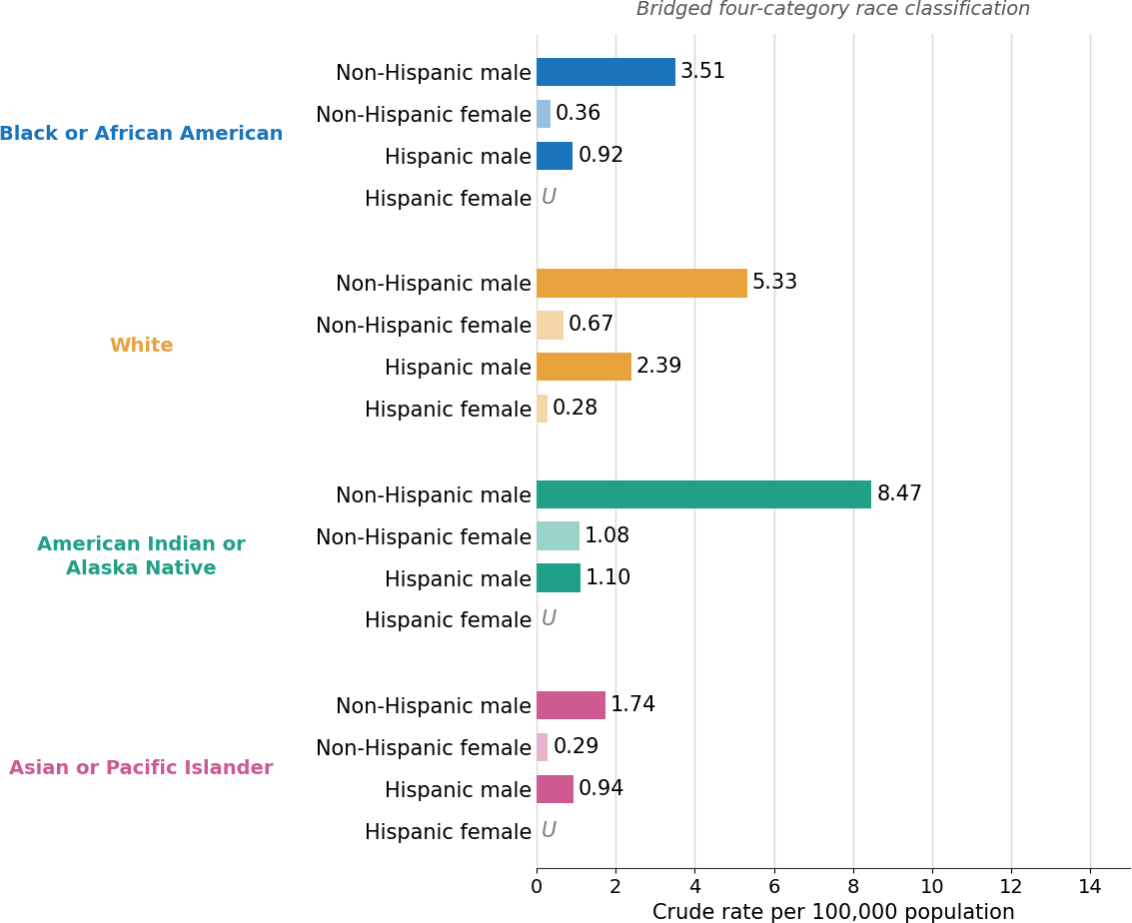
Crude firearm suicide rates among US youth and young adults aged 0 to 24 years, by race, Hispanic ethnicity, and sex, using the bridged 4-category race classification for 1999 to 2020. S indicates suppressed data because the cell contained fewer than 10 deaths; U indicates unreliable data because the estimate was based on fewer than 20 deaths. Source: CDC WONDER [25]. CDC: Centers for Disease Control and Prevention.

**Figure 4.**
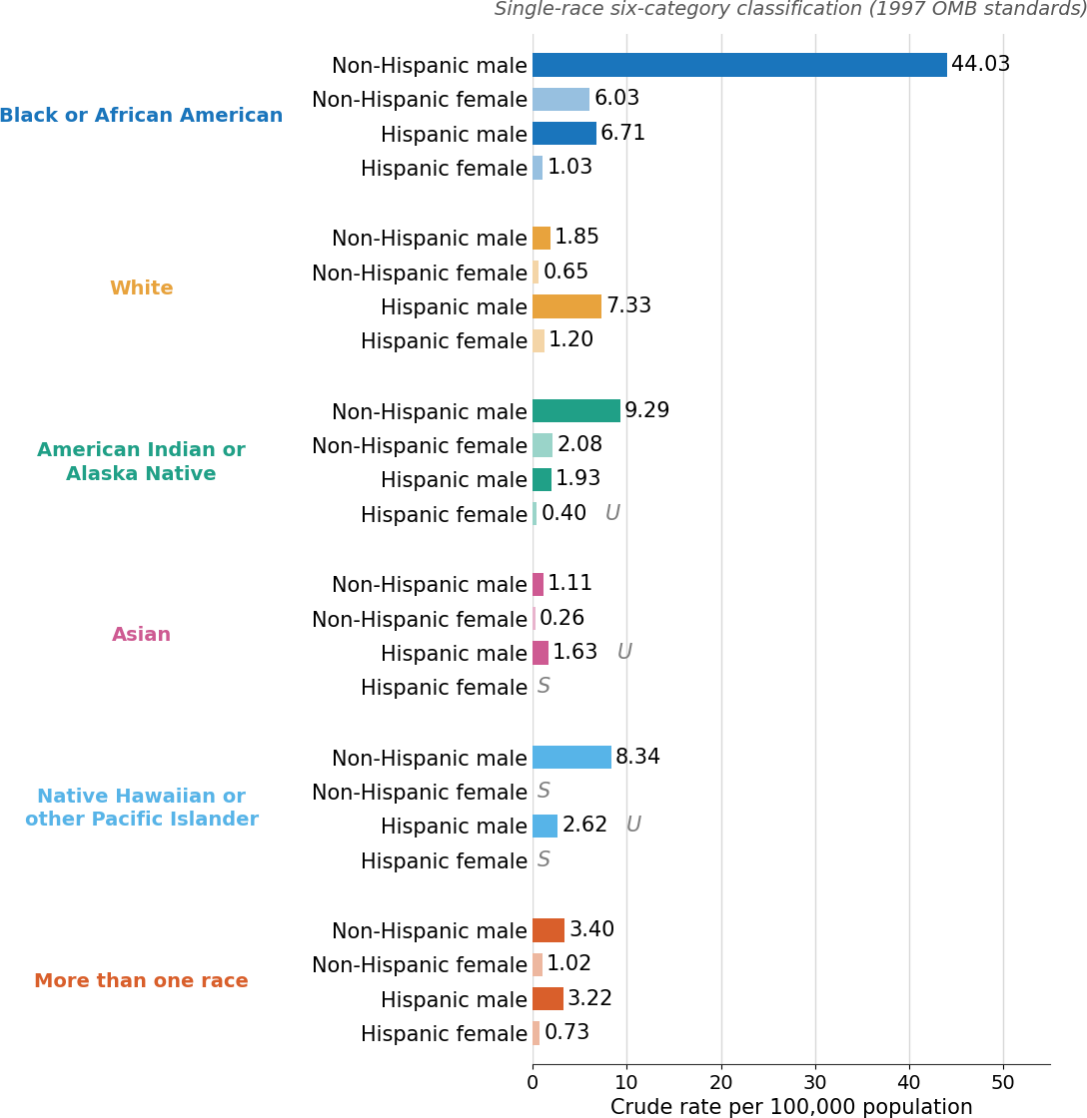
Crude firearm homicide rates among US youth and young adults aged 0 to 24 years, by race, Hispanic ethnicity, and sex, using the single-race 6-category classification for 2018 to 2024. S indicates suppressed data because the cell contained fewer than 10 deaths; U indicates unreliable data because the estimate was based on fewer than 20 deaths. Source: CDC WONDER [25]. CDC: Centers for Disease Control and Prevention; OMB: Office of Management and Budget.

**Figure 5.**
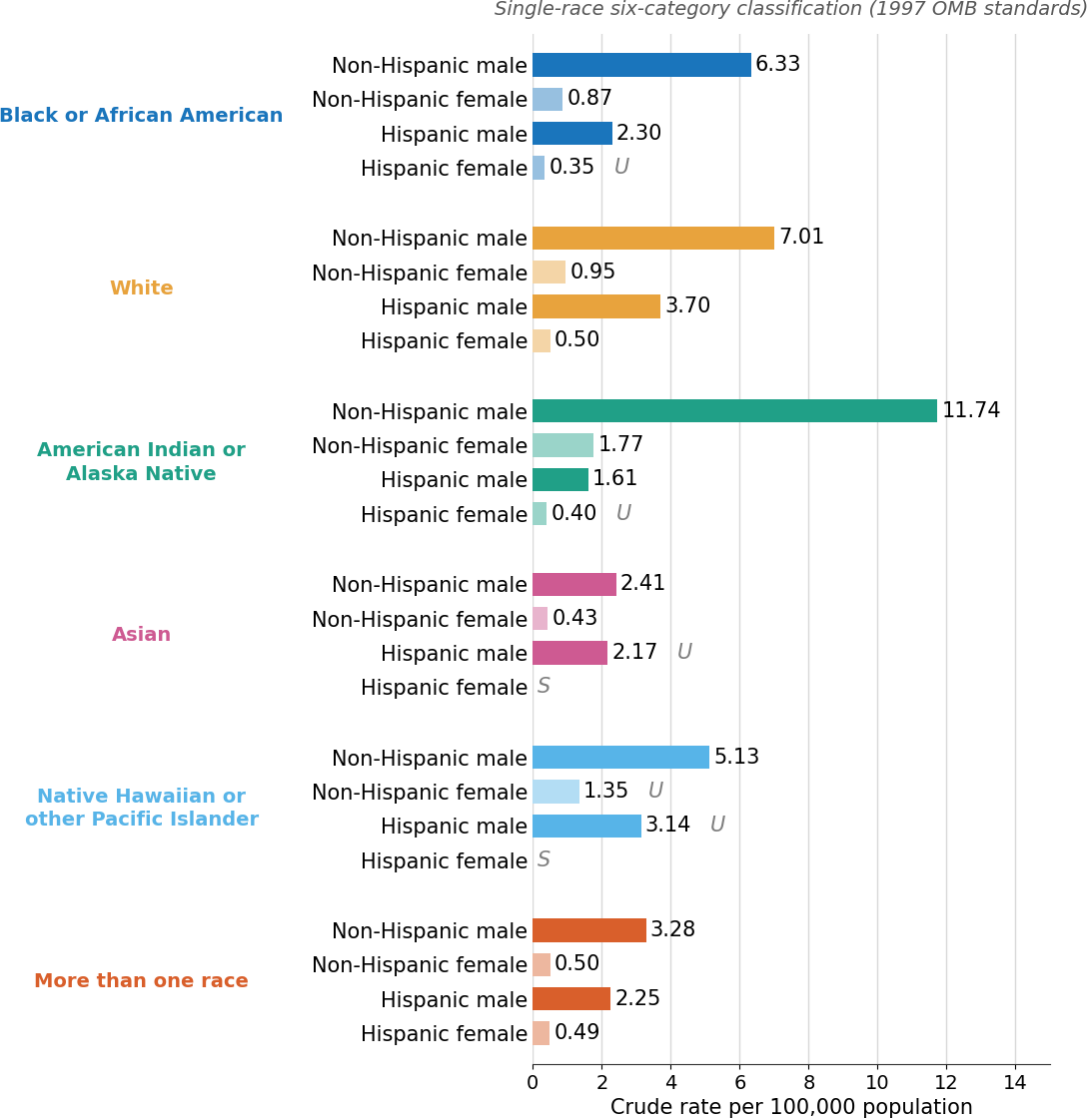
Crude firearm suicide rates among US youth and young adults aged 0 to 24 years, by race, Hispanic ethnicity, and sex, using the single-race 6-category classification for 2018 to 2024. S indicates suppressed data because the cell contained fewer than 10 deaths; U indicates unreliable data because the estimate was based on fewer than 20 deaths. Source: CDC WONDER [25]. CDC : Centers for Disease Control and Prevention.

During period 2, Black non-Hispanic male youth experienced the highest firearm homicide rate of any group examined (44.03 per 100,000; 95% CI 43.46‐44.61), which was 23.8 times the rate among White non-Hispanic male youth (1.85 per 100,000; 95% CI 1.79‐1.92; Figures 2-5; Table 2). AI/AN non-Hispanic male youth exhibited the second-highest rate (9.29 per 100,000; 95% CI 8.20‐10.38), followed by NH/OPI non-Hispanic males (8.34 per 100,000; 95% CI 6.44‐10.64). Among Hispanic-origin male strata, White Hispanic males (7.33 per 100,000; 95% CI 7.14‐7.52) and Black Hispanic males (6.71 per 100,000; 95% CI 6.01‐7.40) carried the highest homicide burdens.

#### Female Homicides

Black non-Hispanic female youth had the highest firearm homicide rate of any female stratum (6.03 per 100,000; 95% CI 5.82‐6.25), 9.3 times the rate among White non-Hispanic female youth (0.65 per 100,000; 95% CI 0.61‐0.69). Notably, this rate exceeded the homicide rates observed in male youth across 7 of the 12 other male groups examined, a pattern typically obscured by sex-aggregated reporting or by race-only stratifications that pool data for Black non-Hispanic girls with other female populations.

### Firearm Suicide Rates by Race or Ethnicity and Sex, 2018-2024

#### Male Suicides

The demographic distribution of firearm suicide differed substantively from that of homicide. AI/AN non-Hispanic male youth experienced the highest firearm suicide rate of any group (11.74 per 100,000; 95% CI 10.51‐12.97), 1.7 times the White non-Hispanic male rate (7.01 per 100,000; 95% CI 6.89‐7.13; Table 2). Black non-Hispanic male youth also showed an elevated rate (6.33 per 100,000; 95% CI 6.11‐6.55), placing them above NH/OPI non-Hispanic males (5.13 per 100,000; 95% CI 3.67‐6.99) and below the AI/AN and White non-Hispanic strata.

#### Female Suicides

Among female youth, AI/AN non-Hispanic females had the highest firearm suicide rate (1.77 per 100,000; 95% CI 1.32‐2.33), followed by NH/OPI non-Hispanic females (1.35 per 100,000; 95% CI 0.65‐2.49, interpret with caution) and White non-Hispanic females (0.95 per 100,000; 95% CI 0.90‐0.99). Black non-Hispanic female firearm suicide rates (0.87 per 100,000; 95% CI 0.79‐0.95), while lower in absolute terms than rates for AI/AN and White non-Hispanic females, showed the steepest within-period relative increase of any female stratum examined (see AAPC analysis in the section below titled “AAPCs in Firearm Homicide and Suicide Rates, 2018-2024”).

### AAPCs in Firearm Homicide and Suicide Rates, 2018-2024

#### Overview

AAPCs were estimated for all non-Hispanic race-sex strata with at least 3 nonsuppressed annual observations between 2018 and 2024 (Table 3), separately for firearm homicide and suicide.

**Table 3.** AAPCs^a^^,^^b^ in crude firearm homicide and suicide mortality rates among US youth and young adults aged 0 to 24 years, by race or ethnicity and sex, non-Hispanic strata, 2018‐2024.

Race/ethnicity group	Sex	Years observed	AAPC % (95% CI)	*P* value	Annual crude rates per 100,000 across years (2018‐2024)
Firearm homicide					
Black NH^c^	Male	7	+2.4% (−7.2 to +13.0)	.56	33.28; 37.10; 52.34; 53.75; 49.74; 45.08; 37.06
Black NH	Female	7	+4.7% (−6.2 to +16.8)	.33	4.35; 4.60; 6.55; 8.10; 7.11; 6.21; 5.31
White NH	Male	7	−1.5% (−8.3 to +5.8)	.61	1.72; 1.68; 2.33; 1.99; 1.93; 1.80; 1.52
White NH	Female	7	+0.1% (−5.3 to +5.8)	.96	0.61; 0.58; 0.77; 0.64; 0.72; 0.64; 0.59
AI/AN^d^ NH	Male	7	+6.4% (−1.0 to +14.3)	.08	7.65; 8.59; 9.07; 6.96; 11.47; 10.70; 10.87
AI/AN NH	Female	4	+10.2% (−9.9 to +34.7)	.17	2.40; 2.40; 2.48; 3.28
Asian NH	Male	7	−2.7% (−14.5 to +10.7)	.61	0.94; 1.17; 1.29; 1.28; 1.01; 1.44; 0.69
Asian NH	Female	—^e^	—	—	Insufficient data
NH/OPI^f^ NH	Male	5	−3.0% (−8.3 to +2.7)	.19	10.37; 9.46; 10.74; 8.87; 8.41
NH/OPI NH	Female	—	—	—	No data
Multiracial NH	Male	7	+5.8% (−2.9 to +15.2)	.15	2.38; 2.92; 3.21; 4.44; 3.89; 3.58; 3.30
Multiracial NH	Female	7	+5.8% (−6.0 to +19.1)	.27	0.84; 0.68; 0.94; 1.51; 0.99; 1.20; 0.96
Firearm suicide					
Black NH	Male	7	+9.2% (+3.2 to +15.4)	.01*	4.68; 4.51; 6.05; 7.28; 7.25; 7.24; 7.28
Black NH	Female	7	+11.7% (−1.7 to +27.0)	.08	0.62; 0.51; 0.68; 1.22; 1.17; 0.81; 1.07
White NH	Male	7	−0.1% (−3.4 to +3.2)	.91	6.98; 6.54; 7.20; 7.92; 6.79; 6.86; 6.80
White NH	Female	7	−0.1% (−3.4 to +3.4)	.95	1.02; 0.88; 0.95; 0.98; 0.86; 1.00; 0.96
AI/AN NH	Male	7	+5.6% (−5.2 to +17.6)	.25	9.01; 8.36; 14.41; 13.23; 14.10; 9.73; 13.59
AI/AN NH	Female	—	—	—	Insufficient data
Asian NH	Male	7	+2.4% (−4.2 to +9.6)	.40	2.02; 2.26; 2.27; 2.98; 2.43; 2.75; 2.17
Asian NH	Female	6	+0.5% (−10.0 to +12.3)	.90	0.48; 0.43; 0.43; 0.42; 0.35; 0.59
NH/OPI NH	Male	—	—	—	No data
NH/OPI NH	Female	—	—	—	No data
Multiracial NH	Male	7	+2.6% (−2.1 to +7.5)	.22	2.86; 3.53; 2.94; 3.52; 3.02; 3.36; 3.72
Multiracial NH	Female	5	+4.0% (−2.5 to +10.9)	.15	0.49; 0.51; 0.46; 0.58; 0.61

a AAPC: average annual percentage change.

b Average annual percentage changes were estimated using log-linear regression of the natural logarithm of the annual crude rate on calendar year.

c NH: non-Hispanic.

d AI/AN: American Indian or Alaska Native.

e Not available. Insufficient nonsuppressed annual observations for estimation.

f NH/OPI: Native Hawaiian or Other Pacific Islander.

#### Firearm Homicides

No non-Hispanic race-sex stratum showed a statistically significant linear trend in firearm homicide across 2018‐2024, reflecting the nonmonotonic trajectories across this window. Among Black non-Hispanic male youth, annual rates rose from 33.28 per 100,000 in 2018 to a peak of 53.75 in 2021, then declined progressively to 37.06 in 2024 (AAPC +2.4%; 95% CI −7.2 to +13.0; *P*=.56). Similar nonmonotonic trajectories appeared among Black non-Hispanic female youth (AAPC +4.7%; *P*=.33) and AI/AN non-Hispanic male youth (AAPC +6.4%; *P*=.08), with each group returning toward prepandemic levels by 2024. White non-Hispanic male rates remained low and stable (AAPC −1.5%; *P*=.61). Despite the relative reversion among the burdened groups, the Black-to-White non-Hispanic male homicide rate ratio increased modestly from 19.3 in 2018 to 24.4 in 2024, indicating that the partial reversion in relative rates was concentrated in the reference group rather than in the Black non-Hispanic male group, and that the relative disparity widened rather than narrowed across the period (Table 3).

#### Firearm Suicides

In contrast to homicide, firearm suicide showed sustained directional trends concentrated in Black non-Hispanic strata. Rates among Black non-Hispanic male youth increased significantly at an AAPC of +9.2% per year (95% CI +3.2 to +15.4; *P*=.01), shifting the Black-to-White non-Hispanic male suicide rate ratio from 0.67 in 2018 to 1.07 in 2024, marking the first year in the 2018 to 2024 series in which the firearm suicide rate among Black non-Hispanic male youth exceeded that among White non-Hispanic male youth. Black non-Hispanic female youth showed a comparable rise in magnitude (AAPC +11.7%; 95% CI −1.7 to +27.0; *P*=.08), falling just outside the conventional threshold for statistical significance and constituting a notable trend warranting close epidemiological surveillance despite the wide CI. White non-Hispanic male and female rates were stable across the same period (AAPC −0.1%; *P*=.91 and *P*=.95, respectively). AI/AN non-Hispanic male suicide rates showed a nonsignificant increasing trend (AAPC +5.6%; 95% CI −5.2 to +17.6; *P*=.25), with substantial year-to-year variability reflecting small population denominators (Table 3).

### Age-Specific Firearm Mortality Patterns

Within period 2, age-specific analysis revealed steep developmental gradients concentrated in 2 intersectional locations (Multimedia Appendix 1). Among Black non-Hispanic male youth, firearm homicide rates increased from 6.63 per 100,000 at ages 10 to 14 years to 86.06 at ages 15 to 19 years and 114.44 at ages 20 to 24 years. The Black-to-White non-Hispanic male homicide rate ratio widened across consecutive age bands, from 26.1 at ages 15 to 19 years to 27 at ages 20 to 24 years, indicating that the greatest absolute firearm homicide burden occurred during late adolescence and young adulthood. This pattern challenges prevention frameworks that define youth as ending at age 17 or 18 because they may exclude young adults aged 18 to 24, including the age group with the highest observed firearm homicide rate.

Among AI/AN non-Hispanic male youth, firearm suicide rates increased from 2.92 per 100,000 at ages 10 to 14 years to 20.16 at ages 15 to 19 years and 31.71 at ages 20 to 24 years. In the oldest age band, the AI/AN non-Hispanic male firearm suicide rate was 1.6 times the corresponding White non-Hispanic male rate (19.61 per 100,000). The parallel adolescent-to-young-adult escalations observed for AI/AN male firearm suicide and Black non-Hispanic male firearm homicide underscore the need for prevention frameworks that extend beyond adolescence into the 18-to-24-year developmental window.

Among female youth, age gradients were less pronounced for homicide but evident for suicide. White non-Hispanic female firearm suicide rates increased from 0.47 per 100,000 at ages 10 to 14 years to 2.40 at ages 20 to 24 years. Black non-Hispanic female firearm suicide rates showed a similar but shallower gradient, increasing from 0.52 to 2.44 per 100,000 across the same age bands. In contrast to the pattern among males, Black and White non-Hispanic female firearm suicide rates across age groups were statistically indistinguishable, with overlapping CIs at ages 10 to 14 and at ages 20 to 24 years, warranting continued monitoring in future surveillance cycles as an indicator of the shifting racial distribution of firearm suicide risk.

## Discussion

### Principal Findings

This 25-year national analysis advances 3 critical findings that aggregate surveillance routinely conceals. Primarily, firearm homicide remains concentrated with extreme disproportionality among Black non-Hispanic male youth and, to a degree not captured by race-only or sex-aggregated reporting, among Black non-Hispanic girls. Annual firearm homicide rates among Black non-Hispanic male youth rose sharply during 2020 and 2021 and substantially reverted by 2024, with the 2024 rate approaching the 2018 level. However, the disparity ratio relative to White non-Hispanic male youth did not narrow across this period. The Black-to-White non-Hispanic male homicide rate ratio increased from 19.3 in 2018 to 24.4 in 2024, indicating that improvement in the absolute homicide rate among Black non-Hispanic males was not accompanied by a reduction in relative inequality.

In addition, firearm suicide burden was highest among AI/AN non-Hispanic male youth and intensified with age, peaking at ages 20 to 24 years. Furthermore, firearm suicide rates among Black non-Hispanic male youth increased at a statistically significant rate of +9.2% per year, with a comparable-magnitude rise among Black non-Hispanic female youth and stable rates among White non-Hispanic male and female youth. Consequently, the Black-to-White non-Hispanic male suicide rate ratio shifted from 0.67 in 2018 to 1.07 in 2024, marking the first year in recent surveillance history that Black non-Hispanic male rates exceeded those of White non-Hispanic male youth. Our findings show that firearm mortality among US youth represents 2 epidemiologically distinct phenomena that require intent-specific, intersectionally disaggregated surveillance and prevention paradigms.

### Comparison With Prior Work

#### Firearm Homicide

The firearm homicide rate among Black non-Hispanic male youth (44.03 per 100,000), 23.8 times that of White non-Hispanic male youth, is consistent with prior evidence linking elevated community violence exposure to structural conditions, not limited to residential segregation, concentrated economic disinvestment, and limited access to opportunity in the particular neighborhoods where these conditions co-occur, rather than to membership in any racial or ethnic group [5,26]. Crucially, this disparity ratio widened further with age, reaching 27.0 per 100,000 at ages 20 to 24 years, indicating that the transition from adolescence to young adulthood represents a critical period of escalating rather than declining risk. This age gradient has direct programmatic implications, as prevention frameworks that treat individuals aged 18 years and older as outside the scope of youth-focused intervention may be systematically misaligned and disconnected from the life stage at which absolute homicide risk peaks. Community violence intervention programs, hospital-based violence interruption, and structural investments in housing stability and economic opportunity represent vital avenues for reducing this burden [27]. However, young adults aged 18 to 24 may therefore fall into a prevention and service gap, aging out of pediatric and adolescent systems without a corresponding pathway into adult violence-prevention services unless they experience a violent injury, encounter the criminal legal system, or live in a community served by a dedicated intervention program.

Notably, the firearm homicide rate among Black non-Hispanic female youth (6.03 per 100,000) exceeded that of male youth in 7 of the 12 other racial and ethnic groups. This pattern may be obscured in surveillance reports that aggregate firearm mortality by race and ethnicity without simultaneous stratification by sex [6,8], underscoring the need to explicitly integrate intersectional metrics into clinical and community risk assessment, prevention programming, and policy.

#### Firearm Suicide

AI/AN non-Hispanic male youth had the highest firearm suicide rate of any group (11.74 per 100,000), 1.7 times that of White non-Hispanic males. The absolute burden was greatest among those aged 20 to 24 years, whose firearm suicide rate reached 31.71 per 100,000. Lethal-means safety counseling and secure storage legislation are associated with reduced firearm suicide risk [28], but universal approaches are likely insufficient for AI/AN youth without culturally grounded multitiered strategies developed with (and led by) AI/AN communities. AI/AN youth live across a wide range of contexts, including reservations, rural nonreservations, urban settings, among others, and effective approaches must reflect these distinct community settings. Tribally led prevention partnerships and collaborations with Urban Indian Health Program partnerships offer important infrastructure for addressing community-defined priorities, including limited behavioral health access, the legacy of historical and ongoing structural trauma, and unequal exposure to firearm-injury risk environments [29]. The high year-to-year rate volatility observed in the AI/AN series further indicates that standard surveillance infrastructure is poorly suited to monitoring risk in numerically small populations with elevated burden, and supports the use of pooled estimates, confidence intervals, and surveillance approaches designed for small populations.

The convergence of Black and White non-Hispanic male firearm suicide rates between 2018 and 2024 represents an emerging disparity signal with direct relevance to prevention planning. As firearm homicide and suicide follow distinct demographic concentration patterns, disaggregating by intent is a prerequisite for prevention systems that can match intervention resources to need [2-5,8,9]. The 2024 overall decline in firearm mortality, the largest single-year decline in the current study period, is encouraging, although rates remained elevated relative to pre-2014 levels, and the trajectories among several subgroups warrant continued monitoring.

### Intersectional Interpretation

Three findings illustrate why an intersectional multi-year lens, rather than race-only or sex-only stratification, is necessary to characterize the firearm mortality burden in this age group. Primarily, the firearm homicide rate among Black non-Hispanic female youth (6.03 per 100,000) exceeded male homicide rates in 7 of 12 other racial and ethnic male subgroups examined, a result that is invisible in sex-aggregated reporting and in race-only reporting that pools Black non-Hispanic girls with girls of all other groups. This is the empirical pattern that intersectional theory predicts, noting that risk concentrates at specific social locations defined by the simultaneous operation of multiple structural axes [14-16]. Moreover, the elevated firearm suicide rate among AI/AN non-Hispanic young adult males (31.71 per 100,000 at ages 20 to 24 y) similarly emerges only when race or ethnicity, sex, age, and intent are considered together, reflecting the joint operation of historical, geographic, and behavioral health system exposures rather than any single attribute [29].

In addition, the convergence of Black and White non-Hispanic male firearm suicide rates between 2018 and 2024 demonstrates that intersectional locations are not static. The rate ratio shifted from 0.67 to 1.07 over 6 years, indicating that the demographic distribution of firearm suicide risk is itself in motion. This shift is reinforced by a parallel rise among Black non-Hispanic female youth (AAPC +11.7%; 95% CI −1.7 to +27.0), which approaches but does not reach the conventional threshold for statistical significance and which is similarly invisible in sex-aggregated or race-only reporting. The sex-symmetric character of the Black non-Hispanic firearm suicide rise is precisely the kind of dynamic that intersectional surveillance can detect and that aggregated reporting cannot. For prevention planning, these findings imply that resources designed around the historical White male suicide profile or the historical Black male homicide profile cannot be assumed to reach the youth populations now bearing the greatest and most rapidly changing burden. Intersectional surveillance must be matched with intersectional intervention design, developed with the affected communities [15,16].

### Surveillance Implications

Several populations with elevated burden in this analysis, including Black non-Hispanic girls, AI/AN youth, and NH/OPI adolescents, remain difficult to characterize reliably in national datasets because of small population denominators, historical racial category aggregation, misclassification of injury intent, and suppression thresholds that remove cells with fewer than 10 deaths [13,30]. Of the 72 possible intersectional cells in period 2, only 42 (58.3%) yielded stable estimates. NH/OPI youth were the most severely affected by suppression, with 10 of 12 (83.3%) cells either suppressed or unreliable, meaning that a population group with documented elevated homicide burden is effectively invisible in standard surveillance outputs. This gap reflects a systemic shortcoming in national mortality surveillance, particularly for populations facing the highest levels of risk.

Addressing these gaps requires action at multiple levels. Vital statistics systems should improve the completeness and accuracy of race and ethnicity classification, particularly for AI/AN, Hispanic, Asian, and multiracial decedents. In addition, supporting tribally led and tribally governed data-linkage initiatives, developed through sovereign-to-sovereign partnerships among Tribal Nations, Tribal Epidemiology Centers, Urban Indian Organizations, the Indian Health Service, and state vital statistics agencies, may help reduce racial misclassification in mortality records and improve identification of AI/AN deaths. Such initiatives must proceed under tribal authority, community-defined governance, formal data-use agreements, and appropriate tribal review, consistent with Indigenous Data Sovereignty and CARE principles (Collective benefit, Authority to control, Responsibility, Ethics). Because linkage primarily improves numerator accuracy rather than the size or stability of the underlying population denominator, it should be paired with multiyear estimates, transparent uncertainty reporting, and improved population-estimation methods for small and geographically diverse AI/AN populations. Linkage cannot, however, resolve the small-denominator problem itself, as the instability of rates in numerically small populations is a function of population size rather than of counting error.

Tribal data sovereignty frameworks that enable AI/AN communities to generate, govern, and analyze their own public health data could also improve the capacity to detect and respond to concentrated burden [30]. Finally, the 2018 race-classification transition, while methodologically necessary, created a discontinuity that has constrained intersectional analyses for nearly a decade. Prospective planning for future classification updates should include explicit transition protocols that preserve analytic continuity (e.g., bridging algorithms), rather than requiring investigators to construct post hoc bridging designs.

National vital statistics surveillance is not the only level at which firearm injury can be counted or addressed. Community-based surveillance systems, including hospital trauma registries, school-based injury reporting, faith-based and neighborhood-level violence interruption networks, and tribal injury surveillance partnerships, may generate higher-resolution and more contextually grounded data than national mortality files in populations where suppression and small denominators limit the utility of vital statistics; the White Mountain Apache surveillance and prevention system offers one such model [29]. Pairing these systems with community-based interventions [27], such as hospital-linked violence interruption programs, school-based behavioral health, and lethal-means counseling [28], could allow detection and response to be matched at the scale where families and providers actually encounter risk, moving prevention science well beyond universal screening and counseling [27,29]. These findings indicate which populations bear concentrated burden, and do not posit that descriptive mortality data can adjudicate these issues; that work requires evaluation designs this analysis does not provide.

### Limitations

This analysis has several limitations. CDC WONDER suppresses strata with fewer than 10 deaths, which limits estimation for some intersectional subgroups. Small denominators among AI/AN, NH/OPI, and multiracial youth increase rate instability. The 2018 race-classification transition prevents direct cross-period comparisons. Additionally, crude rather than age-adjusted rates were used to preserve interpretability within fine-grained strata but may limit comparability with age-adjusted estimates reported elsewhere. AAPC estimates rest on a 7-year annual series (2018‐2024); the short window precludes reliable detection of trend inflection points, and log-linear regression may understate nonmonotonic dynamics such as the pandemic-era homicide surge and subsequent partial reversion. Our use of AAPC assumes a linear trajectory across the 2018‐2024 period. However, firearm homicides in heavily burdened strata followed a distinctly nonmonotonic path (a sharp pandemic-era spike followed by a post-2022 decline). The resulting nonsignificant *P* values reflect this U-shaped deviation rather than a lack of meaningful year-to-year shifts. Future analyses with longer longitudinal windows should use nonlinear modeling to capture these specific inflection points. AAPCs should be interpreted as directional rather than definitive. Our descriptive design does not account for socioeconomic conditions, urbanicity, or state-level firearm violence policies and precludes causal inference. Sex was determined from death certificate records, which did not include gender identity data. Consequently, the analysis could not examine firearm mortality among transgender, nonbinary, and other gender-diverse populations at the intersections of race and ethnicity, age, and gender. Intent and race or ethnicity classification on death certificates are similarly imperfect, particularly for AI/AN, Hispanic, and Asian American or Pacific Islander decedents [13,30]. Geographic variation was not examined, though prior work demonstrates substantial heterogeneity [2,10].

### Conclusions

Twenty-five years of national mortality data show that firearm mortality among US youth follows distinct, intent-specific patterns across race or ethnicity, sex, and age that aggregate surveillance routinely obscures. The post-2020 surge in firearm homicide among Black non-Hispanic male youth has reverted by 2024, with the 2024 rate approaching the 2018 baseline. However, the Black-to-White non-Hispanic male homicide rate ratio widened modestly across the period (from 19.3 in 2018 to 24.4 in 2024), indicating that the partial reversion was concentrated in the reference group rather than in the burdened group, and that the structural drivers producing this disparity did not narrow. Frameworks that interpret the 2024 decline as evidence of progress should attend to this distinction. Concurrently, firearm suicide rates among Black non-Hispanic male and female youth rose at a comparable magnitude while White non-Hispanic rates remained stable, producing the first year in this dataset in which Black non-Hispanic male suicide rates exceeded those of White non-Hispanic males. This study advances 3 contributions to the surveillance literature: (1) a coherent bridging design that links bridged-race and single-race surveillance eras within a single intersectional framework, enabling continued long-term monitoring across the 2018 classification transition; (2) finalized 2024 estimates with explicit attention to nonmonotonic postpandemic dynamics, including parallel computation of homicide and suicide AAPCs that prior partially stratified analyses have not provided; and (3) an empirical case for intersectional surveillance as the unit of analysis appropriate to populations whose burden and trajectory cannot be characterized by race alone or sex alone. Intent-specific, intersectionally disaggregated surveillance and prevention strategies, developed with the affected communities, continue to be the appropriate response [27,29].

### Interpretation Notes for AAPCs

For firearm homicide, the log-linear AAPC understates nonmonotonic dynamics. Annual homicide rates among Black non-Hispanic male youth illustrate this pattern: the rate rose from 33.28 in 2018 to a peak of 53.75 in 2021 (a 61.5% relative increase over 3 years), then declined progressively to 37.06 in 2024 (a 31% relative decline over 3 years). Consequently, the log-linear fit across the overall 7-year window did not reach statistical significance because the pandemic-era increase was partially offset by subsequent declines. The pattern is interpreted as pandemic-era elevation with partial reversal rather than as a sustained directional trend.

For firearm suicide, 2 strata show sustained directional trends consistent with the intersectional pattern described in the main text: Black non-Hispanic male youth (AAPC +9.2%, *P*=.01) and Black non-Hispanic female youth (AAPC +11.7%, *P*=.08, approaching but not reaching the conventional threshold). White non-Hispanic male and female rates are stable, producing the convergence in the Black-to-White non-Hispanic male suicide rate ratio reported in the main text.

## Supplementary material

10.2196/96931 Multimedia Appendix 1 Age-specific firearm homicide and suicide mortality rates and distribution of firearm deaths among US youth and young adults aged 0 to 24 years, 1999 to 2020 and 2018 to 2024.

10.2196/96931 Checklist 1 STROBE checklist.

## Abbreviations

AAPC: average annual percentage change
AI/AN: American Indian or Alaska Native
CDC: Centers for Disease Control and Prevention
CFR: Code of Federal Regulations
*ICD-10*: *International Statistical Classification of Diseases, Tenth Revision*
NCHS: National Center for Health Statistics
NH/OPI: Native Hawaiian or Other Pacific Islander
STROBE: Strengthening the Reporting of Observational Studies in Epidemiology
