# Supplementary material for: Twenty-Five Years of Firearm Homicides and Suicides Among US Children and Young Adults, 1999 to 2024: Intersectional Surveillance Analysis
Source: JMIR Public Health Surveill. 2026 Jul 31;12:e96931. doi: 10.2196/96931 (PMC13429908; doi:10.2196/96931)
Supplement: Multimedia Appendix 1 [file publichealth-v12-e96931-s001.docx]

**Table S1.** Selected age-specific crude firearm homicide and suicide mortality rates, Period 1, 1999 to 2020, bridged-race classification.

| **Race or ethnicity** | **Sex** | **Injury intent** | **Ages 10-14 y** | **Ages 15-19 y** | **Ages 20-24 y** |
| --- | --- | --- | --- | --- | --- |
| Black or African American | Male | Homicide | 2.76 (2.60-2.93) | 52.71 (51.99-53.44) | 94.30 (93.32-95.30) |
| White | Male | Homicide | 0.57 (0.54-0.61) | 5.75 (5.64-5.86) | 8.67 (8.54-8.81) |
| Black or African American | Female | Homicide | Not estimableᵃ | 5.34 (5.11-5.58) | 8.24 (7.95-8.53) |
| White | Female | Homicide | 0.26 (0.23-0.28) | 1.05 (1.01-1.10) | 1.61 (1.55-1.67) |
| American Indian or Alaska Native | Male | Suicide | Not estimableᵃ | 10.95 (9.95-12.02) | 14.97 (13.78-16.24) |
| American Indian or Alaska Native | Female | Suicide | Not estimableᵃ | Not estimableᵃ | Not estimableᵃ |
| Black or African American | Male | Suicide | Not estimableᵃ | 4.51 (4.30-4.73) | 11.08 (10.75-11.43) |
| White | Male | Suicide | 1.00 (0.95-1.05) | 7.43 (7.30-7.55) | 12.96 (12.80-13.13) |
| Black or African American | Female | Suicide | Not estimableᵃ | Not estimableᵃ | Not estimableᵃ |
| White | Female | Suicide | Not estimableᵃ | 1.02 (0.97-1.07) | 1.55 (1.49-1.61) |

Rates are crude deaths per 100,000 population, with 95% CIs in parentheses. Period 1 estimates use bridged-race categories and include all Hispanic origins because Hispanic origin was not available in the age-specific export for this period.

ᵃNot estimable indicates that one or more annual cells were suppressed, preventing calculation of an exact pooled rate.

**Table S2.** Selected age-specific crude firearm homicide and suicide mortality rates, Period 2, 2018 to 2024, single-race classification.

| **Race or ethnicity** | **Sex** | **Injury intent** | **Ages 10-14 y** | **Ages 15-19 y** | **Ages 20-24 y** |
| --- | --- | --- | --- | --- | --- |
| Black or African American | Male | Homicide | 6.63 (6.14-7.15) | 86.06 (84.28-87.85) | 114.44 (112.43-116.48) |
| White | Male | Homicide | 0.57 (0.50-0.66) | 3.30 (3.12-3.48) | 4.24 (4.04-4.44) |
| Black or African American | Female | Homicide | 1.80 (1.55-2.09) | 10.39 (9.78-11.04) | 14.19 (13.48-14.92) |
| White | Female | Homicide | 0.30 (0.24-0.36) | 0.96 (0.87-1.07) | 1.41 (1.29-1.53) |
| American Indian or Alaska Native | Male | Suicide | 2.92 (1.73-4.61) | 20.16 (16.82-23.96) | 31.71 (27.51-36.37) |
| American Indian or Alaska Native | Female | Suicide | Suppressedᵃ | 3.91 (2.50-5.81) | 3.36 (2.08-5.14) |
| Black or African American | Male | Suicide | 1.07 (0.88-1.29) | 8.81 (8.25-9.40) | 20.14 (19.31-21.01) |
| White | Male | Suicide | 2.04 (1.90-2.19) | 10.57 (10.25-10.90) | 19.61 (19.19-20.04) |
| Black or African American | Female | Suicide | 0.52 (0.39-0.69) | 1.17 (0.97-1.40) | 2.44 (2.15-2.76) |
| White | Female | Suicide | 0.47 (0.40-0.54) | 1.51 (1.39-1.64) | 2.40 (2.25-2.55) |

Rates are crude deaths per 100,000 population, with 95% CIs in parentheses. Period 2 estimates are restricted to non-Hispanic youth and use the six-category single-race classification.

ᵃSuppressed indicates fewer than 10 deaths in the pooled cell, per CDC WONDER confidentiality policy.

**Table S3.** Distribution of firearm deaths among US youth and young adults, by sex, injury intent, and year. Panel A. Period 1, 1999 to 2020, bridged-race classification, N=161,313.

| **Characteristic** | **Deaths, No.** | **% of period total** |
| --- | --- | --- |
| **Sex** |  |  |
| Female | 18,459 | 11.4 |
| Male | 142,854 | 88.6 |
| **Injury intent** |  |  |
| Homicide | 99,453 | 61.7 |
| Suicide | 53,333 | 33.1 |
| Unintentional | 4,834 | 3.0 |
| Undetermined intent | 1,906 | 1.2 |
| Legal intervention or operations of war | 1,787 | 1.1 |

Percentages may not sum to 100.0 because of rounding. Counts include homicide, suicide, unintentional, undetermined-intent, and legal-intervention firearm deaths. Deaths by year are reported for Period 2 only, in Panel B.

**Table S4.** Distribution of firearm deaths among US youth and young adults, by sex, injury intent, and year. Panel B. Period 2, 2018 to 2024, single-race classification, N=65,472.

| **Characteristic** | **Deaths, No.** | **% of period total** |
| --- | --- | --- |
| **Sex** |  |  |
| Female | 8,543 | 13.0 |
| Male | 56,929 | 87.0 |
| **Injury intent** |  |  |
| Homicide | 38,809 | 59.3 |
| Suicide | 23,748 | 36.3 |
| Unintentional | 1,417 | 2.2 |
| Undetermined intent | 982 | 1.5 |
| Legal intervention or operations of war | 516 | 0.8 |
| **Year** |  |  |
| 2018 | 7,942 | 12.1 |
| 2019 | 7,943 | 12.1 |
| 2020 | 10,196 | 15.6 |
| 2021 | 10,954 | 16.7 |
| 2022 | 10,146 | 15.5 |
| 2023 | 9,679 | 14.8 |
| 2024 | 8,612 | 13.2 |

Percentages may not sum to 100.0 because of rounding. Counts include homicide, suicide, unintentional, undetermined-intent, and legal-intervention firearm deaths.

**General notes**

Period 1 and Period 2 should not be compared directly and should not be summed. The two periods use different race-classification systems, population denominators, and Hispanic-origin specifications, and they overlap on the years 2018 to 2020, which account for 26,081 deaths counted in both panels.

Ninety-five percent CIs were calculated using the gamma (exact Poisson) method applied to pooled deaths and person-years. These intervals may differ slightly from those displayed by CDC WONDER, which applies a normal approximation to cells with 100 or more deaths.

Race- and ethnicity-specific death counts are not reported in eTable 3 because finely stratified CDC WONDER exports suppress cells with fewer than 10 deaths, preventing complete category totals. Race- and ethnicity-specific burden is presented as crude rates in the main manuscript and in eTables 1 and 2.

Firearm intent categories follow CDC WONDER injury intent definitions. ICD-10 codes are X93 to X95 for firearm homicide and X72 to X74 for firearm suicide.

Source: CDC WONDER Underlying Cause of Death database, finalized data through 2024.
