# Supplementary material for: Twenty-Five Years of Firearm Homicides and Suicides Among US Children and Young Adults, 1999 to 2024: Intersectional Surveillance Analysis
Source: JMIR Public Health Surveill. 2026 Jul 31;12:e96931. doi: 10.2196/96931 (PMC13429908; doi:10.2196/96931)
Supplement: Checklist 1 [file publichealth-v12-e96931-s002.pdf]

STROBE Checklist — Observational Study (Cross-Sectional)

Manuscript: Twenty-Five Years of Firearm Mortality Among US Youth and Young Adults: Intersectional Disparities by Race, Sex, Age, and Injury Intent, 1999–2024

Authors: Chuka Emezue, PhD, MPH, MPA, CHES®; Tipparat Udmuangpia, PhD, MS, RN

Multimedia Appendix 1

Item No.	Recommendation	Location in Manuscript
	**TITLE AND ABSTRACT**	
1	(a) Indicate the study design with a commonly used term in the title or abstract.	Title: "…Intersectional Disparities by Race, Sex, Age, and Injury Intent, 1999–2024"; Abstract Methods: "serial cross-sectional, population-based descriptive analysis"
1	(b) Provide in the abstract an informative and balanced summary of what was done and what was found.	Abstract (Background, Objective, Methods, Results, Conclusions); pp. 1–2
	**INTRODUCTION**	
2	Background/rationale: Explain the scientific background and rationale for the investigation being reported.	Introduction, paragraphs 1–3; pp. 3–4
3	Objectives: State specific objectives, including any pre-specified hypotheses.	Abstract (Objective); Introduction, final paragraph; pp. 2, 4
	**METHODS**	
4	Study design: Present key elements of study design early in the paper.	Methods, Study Design and Data Source; p. 4
5	Setting: Describe the setting, locations, and relevant dates, including periods of recruitment, exposure, follow-up, and data collection.	Methods, Study Design and Data Source: US residents, 50 states and Washington, DC; 1999–2024 (Period 1: 1999–2020; Period 2: 2018–2024); p. 4
6	Participants: (a) Cohort/cross-sectional — give eligibility criteria, and the sources and methods of selection of participants.	Methods, Study Design and Data Source: US residents aged 0–24 years; all firearm deaths captured via death certificates in CDC WONDER; p. 4
7	Variables: Clearly define all outcomes, exposures, predictors, potential confounders, and effect modifiers. Give diagnostic criteria, if applicable.	Methods, Firearm Fatality Classification: ICD-10 codes for homicide (X93–X95), suicide (X72–X74), unintentional (W32–W34), undetermined (Y22–Y24), legal intervention (Y35.0); race/ethnicity, sex, age as stratification variables; pp. 5–6
8	Data sources/measurement: For each variable of interest, give sources of data and details of methods of assessment (measurement).	Methods, Study Design and Data Source: CDC WONDER Underlying Cause of Death database, National Vital Statistics System death certificates; bridged-race and single-race population denominators; pp. 4–5

Item No.	Recommendation	Location in Manuscript
9	Bias: Describe any efforts to address potential sources of bias.	Methods, Statistical Analysis: suppression and unreliability flags noted; crude vs. age-adjusted rate rationale explained; Limitations: race/ethnicity misclassification on death certificates, intent classification limitations; pp. 6–7, 12
10	Study size: Explain how the study size was arrived at.	Methods, Study Design and Data Source: entire national population of firearm decedents aged 0–24 years, 1999–2024; no sample size calculation required for complete population data; p. 4
11	Quantitative variables: Explain how quantitative variables were handled in the analyses. If applicable, describe which groupings were chosen and why.	Methods, Statistical Analysis: age grouped as 0–12, 13–17, 18–24 years per established developmental frameworks; rates per 100,000 population; pp. 6–7
12	(a) Describe all statistical methods, including those used to control for confounding.	Methods, Statistical Analysis: crude death rates, Byar approximation for 95% CIs, disparity ratios, log-linear regression for AAPCs; pp. 6–7
12	(b) Describe any methods used to examine subgroups and interactions.	Methods, Statistical Analysis: simultaneous stratification by race/ethnicity, sex, age, and injury intent; suppression and unreliability reporting for thin strata; pp. 6–7
12	(c) Explain how missing data were addressed.	Methods, Statistical Analysis: CDC WONDER suppression protocol applied (cells <10 deaths suppressed; cells <20 deaths flagged unreliable); 26.4% of Period 2 intersectional cells suppressed; p. 7
12	(d) Cross-sectional study — if applicable, describe analytical methods taking account of sampling strategy.	Methods: complete national vital statistics enumeration; no sampling; population-based denominators from CDC WONDER; p. 4
12	(e) Describe any sensitivity analyses.	Methods, Two-Period Analytic Design: overlapping years 2018–2020 used to evaluate estimate consistency across bridged-race and single-race periods; p. 6
	**RESULTS**	
13	(a) Report numbers of individuals at each stage of the study.	Results, Temporal Trends: 199,452 total firearm deaths (160,589 Period 1; 38,863 non-overlapping Period 2 years); p. 8
13	(b) Give reasons for non-participation at each stage.	N/A — complete national vital statistics enumeration; no participant recruitment or attrition
13	(c) Consider use of a flow diagram.	Not applicable; complete population data used; suppression patterns described in Methods (Statistical Analysis) and Table notes
14	Descriptive data: (a) Give characteristics of study participants and information on exposures and potential confounders.	Results, Temporal Trends and Tables 1–2: rates stratified by race/ethnicity, sex, age, and injury intent for both periods; pp. 8–10

Item No.	Recommendation	Location in Manuscript
14	(b) Indicate number of participants with missing data for each variable of interest.	Methods, Statistical Analysis and Results: 19 of 72 Period 2 intersectional cells suppressed (<10 deaths); 11 cells unreliable (<20 deaths); p. 7
15	Outcome data: Report numbers of outcome events or summary measures.	Results, all subsections; Tables 1–2: crude rates with 95% CIs by race/ethnicity, sex, and injury intent for Periods 1 and 2; pp. 8–11
16	Main results: (a) Give unadjusted estimates and, if applicable, confounder-adjusted estimates and their precision.	Results: crude rates and disparity ratios with 95% CIs; AAPCs with 95% CIs and P values; Tables 1–2; Figure 3; pp. 8–11
16	(b) Report category boundaries when continuous variables were categorized.	Methods, Study Design and Data Source: age groups 0–12, 13–17, 18–24 years; p. 4
16	(c) If relevant, consider translating estimates of relative risk into absolute risk for a meaningful time period.	Results: crude rates per 100,000 population (absolute) reported alongside disparity ratios (relative); pp. 8–11
17	Other analyses: Report other analyses done — e.g., subgroup analyses, sensitivity analyses.	Methods, Two-Period Analytic Design: cross-period consistency check using overlapping 2018–2020 years; Results, Age-Specific Patterns; p. 6, 11
	**DISCUSSION**	
18	Key results: Summarise key results with reference to study objectives.	Discussion, Principal Findings; p. 11
19	Limitations: Discuss limitations of the study, taking into account sources of potential bias or imprecision. Discuss both direction and magnitude of any potential bias.	Discussion, Limitations; p. 12
20	Interpretation: Give a cautious overall interpretation of results considering objectives, limitations, multiplicity of analyses, results from similar studies, and other relevant evidence.	Discussion, Comparison With Prior Work; Surveillance Implications; Conclusions; pp. 11–13
21	Generalisability: Discuss the generalisability (external validity) of the study results.	Discussion, Limitations: complete national vital statistics data; generalisable to US resident population but geographic variation not examined; p. 12
	**OTHER INFORMATION**	
22	Funding: Give the source of funding and the role of the funders for the present study and, if applicable, for the original study on which the present article is based.	Acknowledgments: no external funding received; p. 13

Note: N/A = not applicable. Items marked N/A reflect study design features (complete national vital statistics enumeration) that preclude participant flow or non-participation reporting. AAPC = average annual percentage change. CI = confidence interval. CDC = Centers for Disease Control and Prevention. ICD-10 = International Classification of Diseases, Tenth Revision.
